# BRCA1 protein expression and subcellular localization in primary breast cancer: Automated digital microscopy analysis of tissue microarrays

**DOI:** 10.1371/journal.pone.0184385

**Published:** 2017-09-01

**Authors:** Abeer M. Mahmoud, Virgilia Macias, Umaima Al-alem, Ryan J. Deaton, Andre Kadjaksy-Balla, Peter H. Gann, Garth H. Rauscher

**Affiliations:** 1 College of Applied Health Sciences, University of Illinois at Chicago, Chicago, Illinois, United States of America; 2 Department of Pathology, College of Medicine, University of Illinois at Chicago, Chicago, Illinois, United States of America; 3 Department of Pathology, South Egypt Cancer Institute, Assiut University, Assiut, Egypt; 4 Division of Epidemiology and Biostatistics, School of Public Health, University of Illinois at Chicago, Chicago, Illinois, United States of America; University of Alabama at Birmingham, UNITED STATES

## Abstract

**Purpose:**

Mutations in BRCA1 are associated with familial as well as sporadic aggressive subtypes of breast cancer, but less is known about whether BRCA1 expression or subcellular localization contributes to progression in population-based settings.

**Methods:**

We examined BRCA1 expression and subcellular localization in invasive breast cancer tissues from an ethnically diverse sample of 286 patients and 36 normal breast tissue controls. Two different methods were used to label breast cancer tissues for BRCA1: (1) Dual immunofluoresent staining with BRCA1 and cytokeratin 8/18 and (2) immunohistochemical staining using the previously validated MS110 mouse monoclonal antibody. Slides were visualized and quantified using the VECTRA Automated Multispectral Image Analysis System and InForm software.

**Results:**

BRCA1 staining was more intense in normal than in invasive breast tissue for both cytoplasmic (p<0.0001) and nuclear (p<0.01) compartments. BRCA1 nuclear to cytoplasmic ratio was higher in breast cancer cells than in normal mammary epithelial cells. Reduced BRCA1 expression was associated with high tumor grade and negative hormone receptors (estrogen receptor, progesterone receptor and Her2). On the other hand, high BRCA1 expression correlated with basal-like tumors (high CK5/6 and EGFR), and high nuclear androgen receptor staining. Lower nuclear to cytoplasmic ratio of BRCA1 correlated significantly with high Ki67 labeling index (p< 0.05) and family history of breast cancer (p = 0.001).

**Conclusion:**

Findings of this study indicate that alterations in BRCA1 protein expression and subcellular localization in breast cancer correlate with poor prognostic markers and aggressive tumor features. Further large-scale studies are required to assess the potential relevance of BRCA1 protein expression and localization in routine classification of breast cancer.

## Introduction

Breast cancer is the most common cancer and second leading cause of cancer death among women in the United States. In 2015, the American Cancer Society estimated 231,840 new cases of breast cancer and 40,290 breast cancer deaths [1]. Approximately 10% of these cases are likely to be hereditary, and roughly 40–50% of hereditary breast cancer cases are attributed to mutations in BRCA1 gene [2]. BRCA1 helps repair DNA damage and ensures genetic stability as it shuttles between the cytoplasm and the nucleus. Also, BRCA1 exhibits other regulatory aspects in transcriptional activation, cell cycle progression, and chromosomal remodeling [3]. BRCA1 mutations are one of the most established risk factors for breast cancer, resulting in loss of BRCA1 protein function, reduced expression or disturbed subcellular distribution of the protein [4]. Women with a functional BRCA1 mutation have up to an 80% risk of developing breast cancer [5]. In addition to its well-established role in hereditary breast cancer, BRCA1 has more recently been shown to be involved in sporadic breast and ovarian cancer [6]. BRCA1 promoter hypermethylation or overexpression of the BRCA1-targeting micro RNAs (e.g., mir-182) are thought to be mechanisms of downregulation of BRCA1in sporadic cancers [7].

In addition to its expression, BRCA1 subcellular localization is an important contributor to its function [8]. There are significant discrepancies in clinical data regarding the nuclear/cytoplasmic distribution of BRCA1 in breast cancer [4, 9–11] and lack of knowledge regarding whether BRCA1 expression level or its nuclear localization is the primary contributing factor to breast cancer development and progression. Inconsistent results concerning the association of BRCA1 expression with cancer progression may reflect differences in the quality of BRCA1 protein quantification. In the present study, we evaluated BRCA1 protein expression and cellular localization via immunohistochemical staining in a well-characterized series of breast cancer patients using MS110 monoclonal antibody that has been validated in previous studies. Furthermore, we used dual immunofluorescence (IF) staining with BRCA1 and cytokeratin 8/18 to identify BRCA1 staining in the epithelial versus the stromal compartments along with the nuclear DAPI stain in order to detect the subcellular localization of BRCA1. BRCA1 expression and compartmental and subcellular localization were evaluated using VECTRA automated digital analysis system. BRCA1 cytoplasmic and nuclear expression was then correlated with age, race, family history, clinicopathological characteristics such cancer stage, histological grade and subtype, molecular subtype, basal cell nature, and other prognostic markers such as Ki67, P53, Bcl2, and androgen receptor (AR).

## Materials and methods

### Study population and biological samples

Patients and samples come from the “Breast Cancer Care in Chicago” study, a population-based cross-sectional study of primary in-situ or invasive breast cancer diagnosed between October 2005, and February 2008. We obtained paraffin-embedded surgical samples of the tumor before initiation of any radiation, chemotherapy or hormone therapy. Clinical histories and tumor characteristics including stage at diagnosis, histology and grade were abstracted from medical records. All participants provided written informed consent. The parent study protocol, subsequent tissue-based analyses and consent forms were approved by the University of Illinois at Chicago Institutional Review Board which have been previously published [12].

### Construction of the tissue microarray

Tissues microarrays (TMAs) were constructed from 286 breast cancer patients, 36 normal breast tissue sections from unaffected women obtained by reduction mastectomy procedures from UIC Medical Center. For each case, a representative area of invasive breast cancer was identified by a trained pathologist on hematoxylin and eosin stained sections and marked on individual paraffin blocks for the creation of TMA. TMAs were prepared as previously described [13]. In brief, triplicate, 0.6-mm-diameter tissue cores were punched from representative tumor regions of each donor block and arrayed into a new recipient paraffin block using a tissue microarrayer (Beecher Instruments, Silver Spring, MD). TMA blocks were constructed in triplicates, each containing one sample from a different region of the tumor.

### Immunohistochemical and immunofluorescent staining

Serial sections from the tissue microarrays (4 μm) were cut, deparaffinized, rehydrated, and subjected to the appropriate antigen retrieval and non-specific binding blocking methods. Sections were then incubated with the appropriate primary and secondary antibody and visualized with 3,3-diaminobenzidine (DAB) and hematoxylin (counterstain). Immunohistochemical (IHC) staining performed by the UIC Histology Core Facility was optimized by testing different sources and dilutions of the primary antibody, and different methods of antigen retrieval. The staining for estrogen receptor (ER), progesterone receptor (PR), Her2, EGFR, P53, and bcl2 were performed at the Medical Center at Chicago Clinical Reference Surgical Pathology laboratory using clinically validated antibodies and standard IHC staining procedures. The staining for BRCA1, androgen receptor (AR), cytokeratin 5/6, and Ki67 was performed at the University of Illinois Research Histology and Tissue Imaging Core. For BRCA1 IHC staining, MS110 mouse monoclonal antibody was used at a dilution of 1:200. For BRCA1 dual immunofluorescent (IF) staining, tissues were incubated with polyclonal rabbit anti-BRCA1 antibody (Cat. # HPA034966, Sigma-Aldrich) and the CK8/18 antibody (American Research Products), both at a titer of 1:100 for 60 minutes at room temperature. After washing with TBS, sections were incubated with the secondary antibodies, anti-mouse Alexa Fluor 647 and anti-rabbit Alexa Fluor 488 polymer, for 20 minutes at room temperature. Slides were rinsed in distilled water; nuclei were counterstained with DAPI. Positive and negative controls were included in each assay series. A list of antibodies for IHC and IF staining is summarized in Table 1.

**Table 1:** List of antibodies for immunofluorescent and immunohistochemical staining.

Antigen	Manufacturer	Host	Clone #	Dilution	Retrieval method
BRCA1-IF	Sigma-Aldrich	Rabbit	Polyclonal	1:100	HIER
BRCA1-IHC	Thermoscientific	Mouse	MS110	1:200	HIER
P53	Ventana	Mouse	BP-53-11	Predilute	CC1 Mild
Ki67	Abcam	Rabbit	SP6	1:100	HIER
Bcl2	CellMarque	Mouse	124	Predilute	CC1 Mild
AR	DAKO	Mouse	AR441	1:50	CC1 Mild
ER	Ventana	Rabbit	SP1	Predilute	CC1 Mild
PR	Ventana	Rabbit	100	Predilute	CC1 Mild
Her-2	Ventana	Mouse	4B5	Predilute	CC1 Mild
CK 5/6	DAKO	Mouse	D5 & 16B4	1:50	HIER
EGFR	Ventana	Mouse	3C6	Predilute	CC1 Mild

HIER, Heat-induced epitope retrieval; CC1, cell conditioning solution 1

### Evaluation of staining

**Fig 1:**
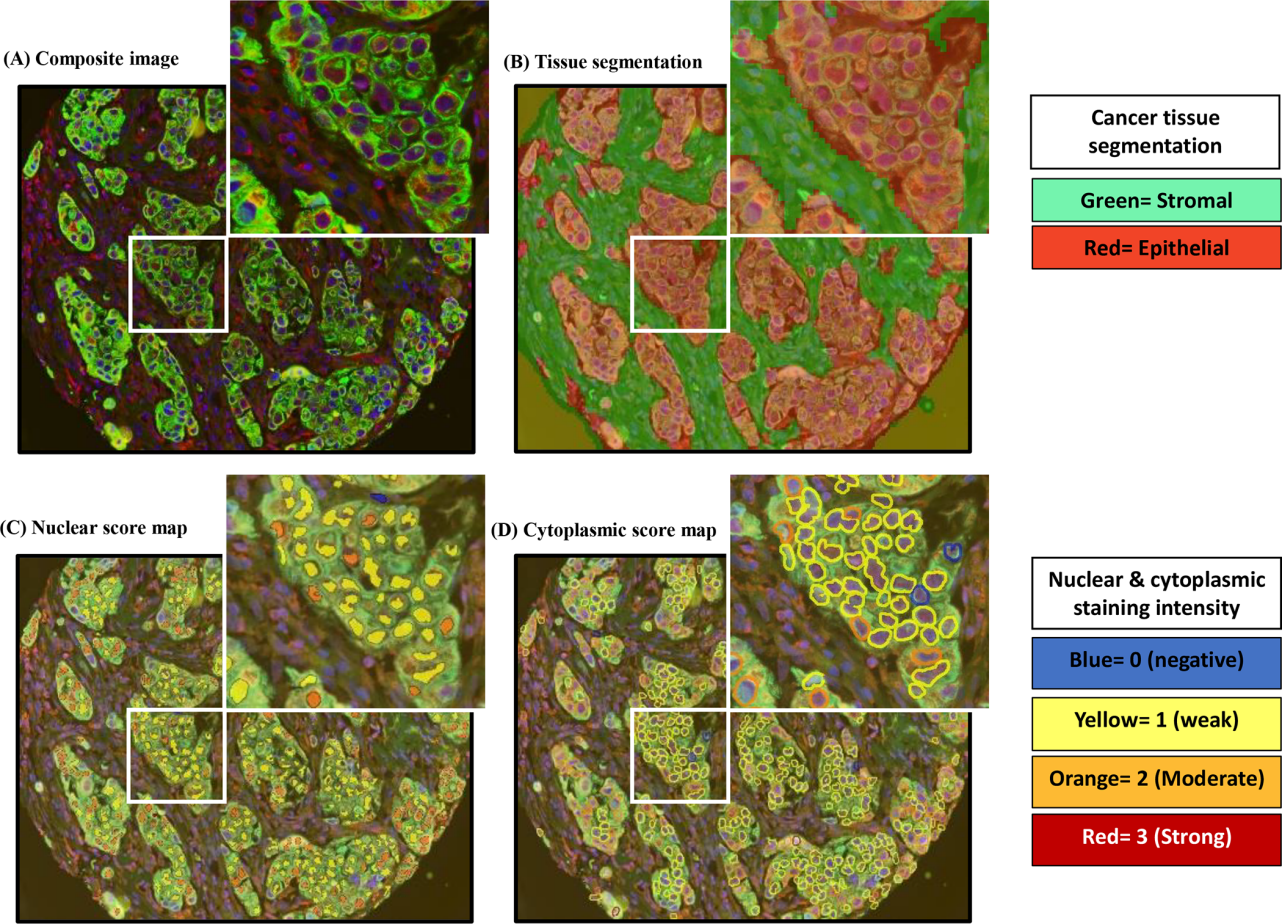
Dual IF staining for BRCA1 and CK8/18 and corresponding digital annotation for BRCA1 in a representative core. A: Composite image where green fluorescent staining if for epithelial CK8/18, red is for BRCA1, and blue is for nuclear DAPI stain. B: Tissue segmentation. C: Nuclear score map. D: Cytoplasmic score map. The attached legend to the right indicates what each color stands for in the tissue segmentation and score maps.

**Fig 2:**
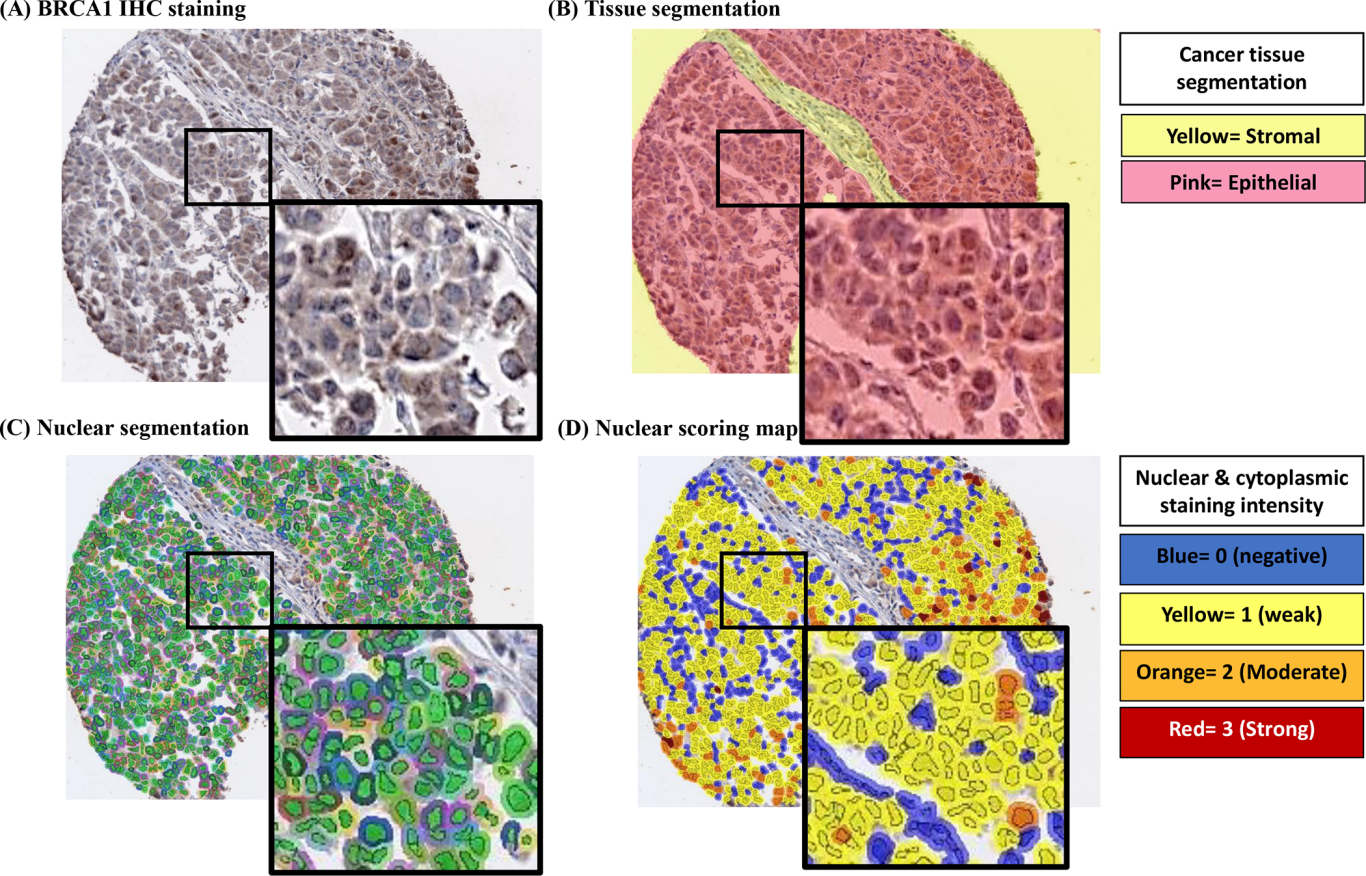
IHC staining and corresponding digital annotation for BRCA1 in a representative core. A: IHC staining utilizes MS110 mouse monoclonal antibody to assess the level of BRCA1 protein expression. B: Tissue segmentation where the epithelial compartment is pink and the stromal compartment is yellow. C: Nuclear segmentation. D: nuclear score map. Definition of the digital image annotation intensity score: blue = 0, yellow = 1, orange = 2 and red = 3. The attached legend to the right indicates what each color stands for in the tissue segmentation and score map.

For digital analysis of the dual IF (Fig 1) and IHC (Fig 2) stained TMAs, the Vectra® (PerkinElmer) multispectral image analysis system was used. Stained slides were scanned with the multispectral Vectra scanner and quantitative imaging system (Perkin Elmer, Hopkinton, MA). For the dual IF staining, CK8/18 staining was used to identify epithelial versus stromal tissue in the TMAs. InForm v2.0 machine learning algorithms (Perkin Elmer) were used to segment tissue compartments (epithelium vs. stromal) and subcellular compartments (nucleus versus cytoplasm). The outcome of tissue segmentation (epithelium versus stroma) for each core image was assessed by a trained pathologist and manual editing removed benign epithelium from the analysis. DAPI and hematoxylin staining was recognized by the software as the nucleus of each cell in IF and IHC stained slides, respectively and the cytoplasmic signal was obtained by sampling the peri-nuclear area. BRCA1 expression was quantified within the selected tissue and subcellular compartment(s) of interest. Within the tumor areas, we exported both nuclear and cytoplasmic data from the BRCA1 channel on a per-cell basis. BRCA1 expression was evaluated based on the percentage of positive cells and staining intensity using the H-score. The H score is a product of the percentage of cells (0–100%) in each intensity category (0, 1+, 2+ and 3+). The final score is on a continuous scale between 0 and 300.

Immunohistochemical staining and manual scoring for ER, PR, Her2/neu, EGFR and CK5/6 was performed by a trained pathologist without knowledge of case outcomes after a consensus was reached about cut off levels with an experienced pathologist behind a multi-headed microscope. Samples were scored as positive for ER or PR, when 10% or more of tumor cell nuclei showed positive staining for the ER or PR, respectively. For HER2 and EGFR, American Society of Clinical Oncology/College of American Pathologists (ASCO/CAP) guideline recommendations for human epidermal growth factor receptor two testing in breast cancer were used with a membrane-staining score ranging from 0 to +3 [14]. Briefly, a score of zero has no staining, 1+ has 10% of cells or less with faint, barely perceptible incomplete cell membrane staining, 2+ has at least 10% of cells with complete, weak to moderate cell membrane staining and 3+ has at least 10% of cells with circumferential, complete and intense membrane staining. HER2 or EGFR was considered positive when the score was +3. The CK 5/6 was scored as 0 (negative), R (rare; single cells stain), 1+ (5–30% cells stain), 2+ (31–60% cells stain) and 3+ (more than 60% of cells stain) [15]. Any staining (1+ to 3+) was considered to be a positive result for CK5/6. From these results, breast cancers were classified as Luminal A (ER+/PR+/HER2-), Luminal B (ER+/PR+/HER2+), HER2 enriched (ER-/PR-/HER2+), and triple negative (ER-/PR-/HER2-). Triple negative tumors were subclassified into basal-like if they expressed either EGFR or CK5/6 or otherwise classified as unspecified if negative for both EGFR and CK5/6. AR expression was evaluated based on the percentage of positive tumor cells and staining intensity using the H-score method described above. AR expression was then classified as low or high using the mean of the AR score as a cutoff. For Ki67 and P53 the percentage of positively stained cells were quantified in each core which ranged from 0 to 100%. Ki67 labeling index (LI) was classified as low (<14%) or high (≥14%). The percentage of P53 positive cells was also categorized as low (<20%) or high (≥20%). For Bcl-2 scoring a semiquantitative scale was used, which grades the tumors from 0 to 5 depending on the percentage of tumor cells stained and the intensity and homogeneity of the reaction, where 0 = totally negative, 1 = Heterogeneous, <20% of the cells show a reliable staining, 2 = Diffuse, all or most cells show a very light staining, 3 = Heterogeneous, 20–80% show strong staining, 4 = most or all cells show an intermediate but unmistakable positive staining, and 5 = all cells are strongly positive. The semiquantitative score of bcl2 was further classified into negative (scores 0, 1, and 2) and positive (scores 3, 4, and 5)

### Statistical analysis

Our primary outcome variables are stage at diagnosis, histologic grade and molecular subtype (Luminal A, Luminal B, HER2+ and triple negative) according to the expression of ER, PR, HER2, CK 5/6 and EGFR. Stage at diagnosis was categorized using the American Joint Committee on Cancer (AJCC) categories of 0, 1, 2, and 3 and 4. Later stage at diagnosis was defined as stage 2, 3, four vs. 0, 1. Histologic grade was assigned as low, intermediate and high, and categorized as intermediate and high versus low for some analyses. BRCA1 nuclear and cytoplasmic expression was evaluated as continuous scores (scale: 0–300) and as categorical scores (Low: versus high) dichotomized by its median level. We compared mean BRCA1 expression across patient demographic, clinical and tumor characteristics `using Mann-Whitney U tests.

## Results

**Fig 3:**
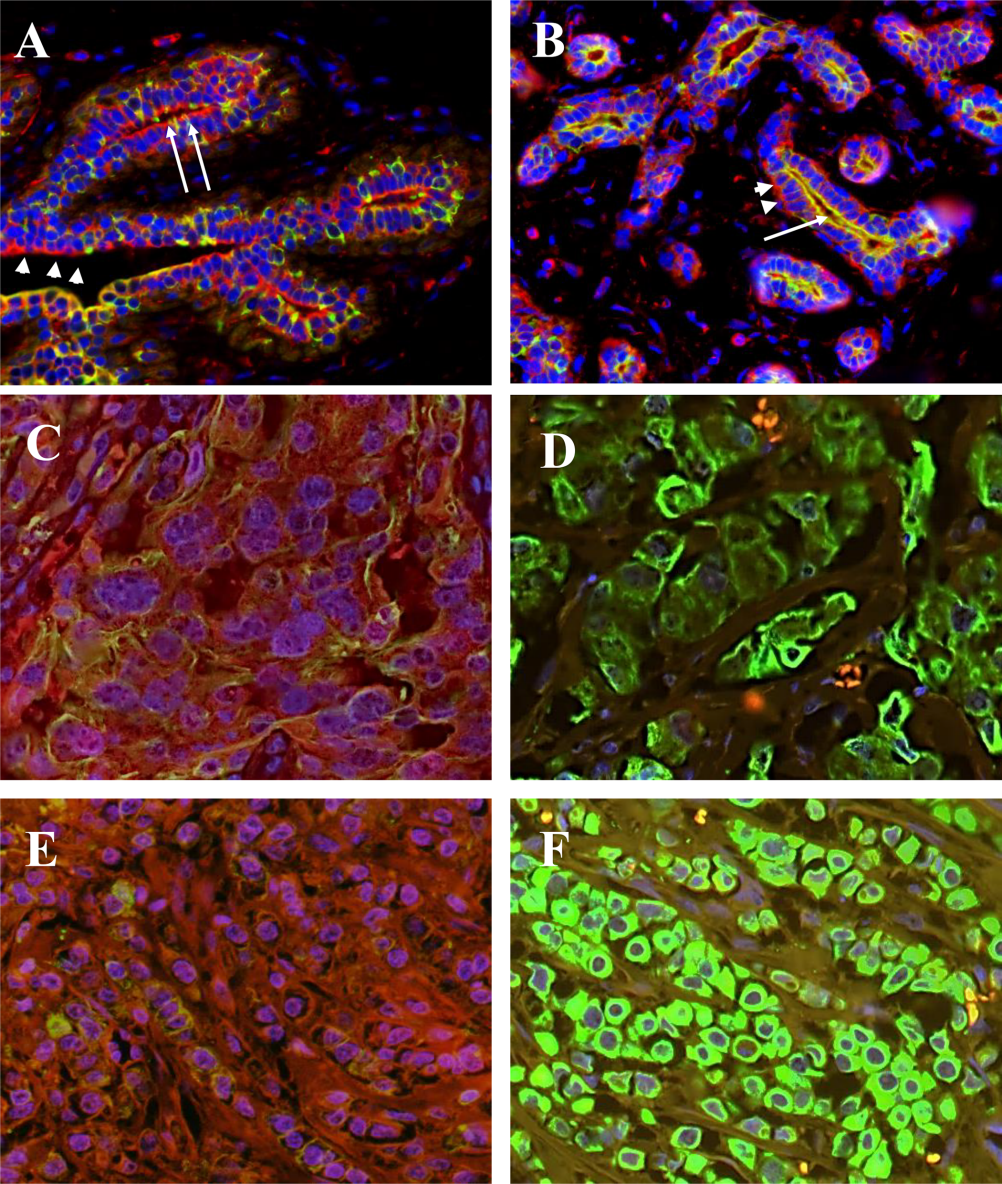
Dual IF staining for BRCA1 and CK8/18 in representative cases of invasive breast cancer and normal breast tissues. A and B: Normal breast ducts and glands with positive red IF for BRCA1 in myoepithelial cells (arrowheads) and luminal cells (arrows). C: BRCA1 positive ductal carcinoma. D: BRCA1 negative ductal carcinoma. E: BRCA1 positive lobular carcinoma. F: BRCA1 negative lobular carcinoma.

BRCA1 protein expression was available for 267 cases (106 nH Black, 80 nH White and 81 Hispanic). Descriptive statistics of this subset are summarized in Table 2. Mean age at diagnosis was 56 years (SD±11). The majority of the cases were of the ductal histological type (76%), luminal A molecular subtype (69%), ER and/or PR-positive (77%), and low or intermediate grade (62%). By the naked eye examination, BRCA1 was strongly and uniformly expressed in the luminal and myoepithelial cell layers of normal mammary glands and ducts (Fig 3A and 3B). In cancer tissues, there was huge inter-tumoral heterogeneity of patterns of cytoplasmic and nuclear BRCA1 staining that ranged from strong positivity (Fig 3C and 3E) to complete absence of staining (Fig 3D and 3F).

**Table 2:** Distribution of the demographic and tumor-related factors for patients included in this tissue microarray study.

BCCC cohort characteristics	N	%
Race/ethnicity (n = 280)		
Black	113	40
nH White	84	30
Hispanic	83	30
Age at diagnosis (n = 233)		
<50	74	32
50+	159	68
Family Breast Cancer <50 (n = 231)		
No	217	94
Yes	14	6
Menopausal (n = 232)		
No	38	16
Yes	194	84
Body Mass Index (n = 278)		
Normal weight	58	21
Overweight or Obese	220	79
Stage at diagnosis (n = 268)		
Early (0,1)	109	41
Late (2,3,4)	159	59
Histologic grade (n = 273)		
Low/intermediate	167	61
High	106	39
ER/PR status (n = 233)		
ER and/or PR-Positive	179	77
Double negative	54	23
Histology (n = 266)		
Ductal	200	75
Lobular	32	12
Mixed ductal/ lobular	19	7
Other	15	6
Molecular Subtypes (n = 266)		
Luminal A	181	68
Luminal B	14	5
Triple Negative	49	19
Her2 enriched	22	8
Basal-like (278)		
No	164	59
Yes	114	41

### Description of BRCA1 expression and subcellular localization in normal and cancer tissue

#### IF staining

**Fig 4:**
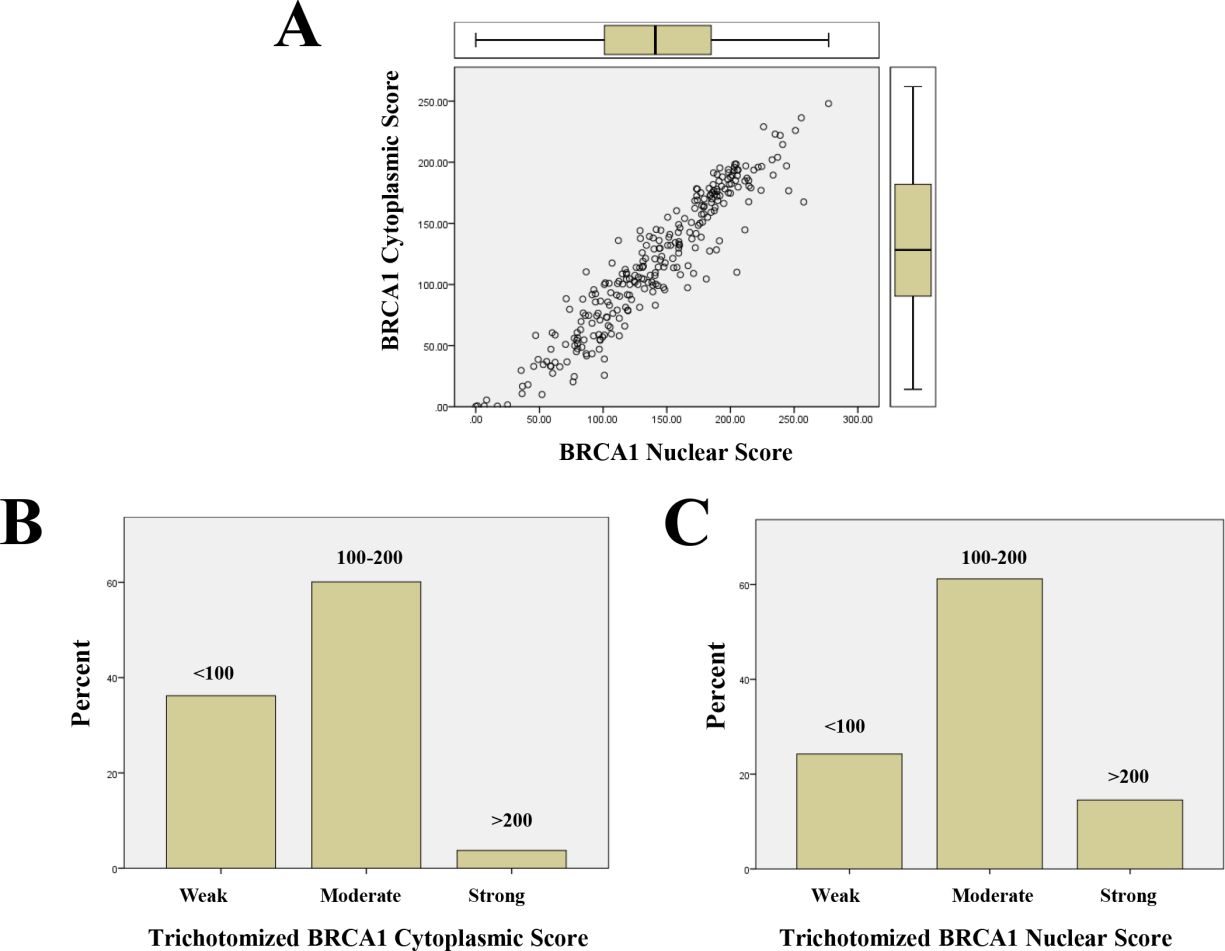
Summary of the nuclear and cytoplasmic BRCA1 staining. A: A plot of pairwise nuclear versus cytoplasmic continuous digital H-score for BRCA1 showing a good correlation between the two locations. B: Percentage of cases that exhibit weak (H-score = <100), Intermediate (H-score > 100 and <200), and strong (H-score >200) cytoplasmic BRCA1 staining. C: Percentage of cases that exhibit weak, Intermediate, and strong nuclear BRCA1 staining.

Digital scoring showed that BRCA1 staining was more intense in normal than in invasive breast tissue for both cytoplasmic (p<0.0001) and nuclear (p<0.01) compartments (Table 3). In normal breast tissue samples, BRCA1 expression was modestly higher in the nucleus than the cytoplasm (156 ± 6 versus 151 ± 8, respectively; p = 0.02, N/C ratio = 1.15±0.1) while in invasive breast cancer samples, a higher BRCA1 expression in the nucleus than in the cytoplasm was more pronounced (141 ± 3 versus 118 ± 3, respectively, p<0.0001, N/C ratio = 1.5±0.1). Yet, there was a strong positive correlation between BRCA1 expression for nuclear and cytoplasmic compartments (r = 0.95, p< 0.0001) (Fig 4A). Weak BRCA1 expression (H-score<100) was less likely in nuclear than cytoplasmic compartments (24% versus 36%, respectively) whereas strong BRCA1 expression (H-score>200) was more likely in nuclear than cytoplasmic compartments (15% versus 4%, respectively) (Fig 4B and 4C).

**Table 3:** Summary of nuclear, cytoplasmic and N/C ratio of BRCA1 expression in normal vs. invasive breast tissue.

		Nuclear Score		Cytoplasmic score		Nuclear/Cytoplasmic	
Variable	N	Mean	P-value	Mean	P-value	Mean	P-value
Breast tissue type			<0.01		<0.0001		<0.0001
Normal	286	158		151		1.2	
Invasive	36	141		118		1.5	

#### IHC staining

**Fig 5:**
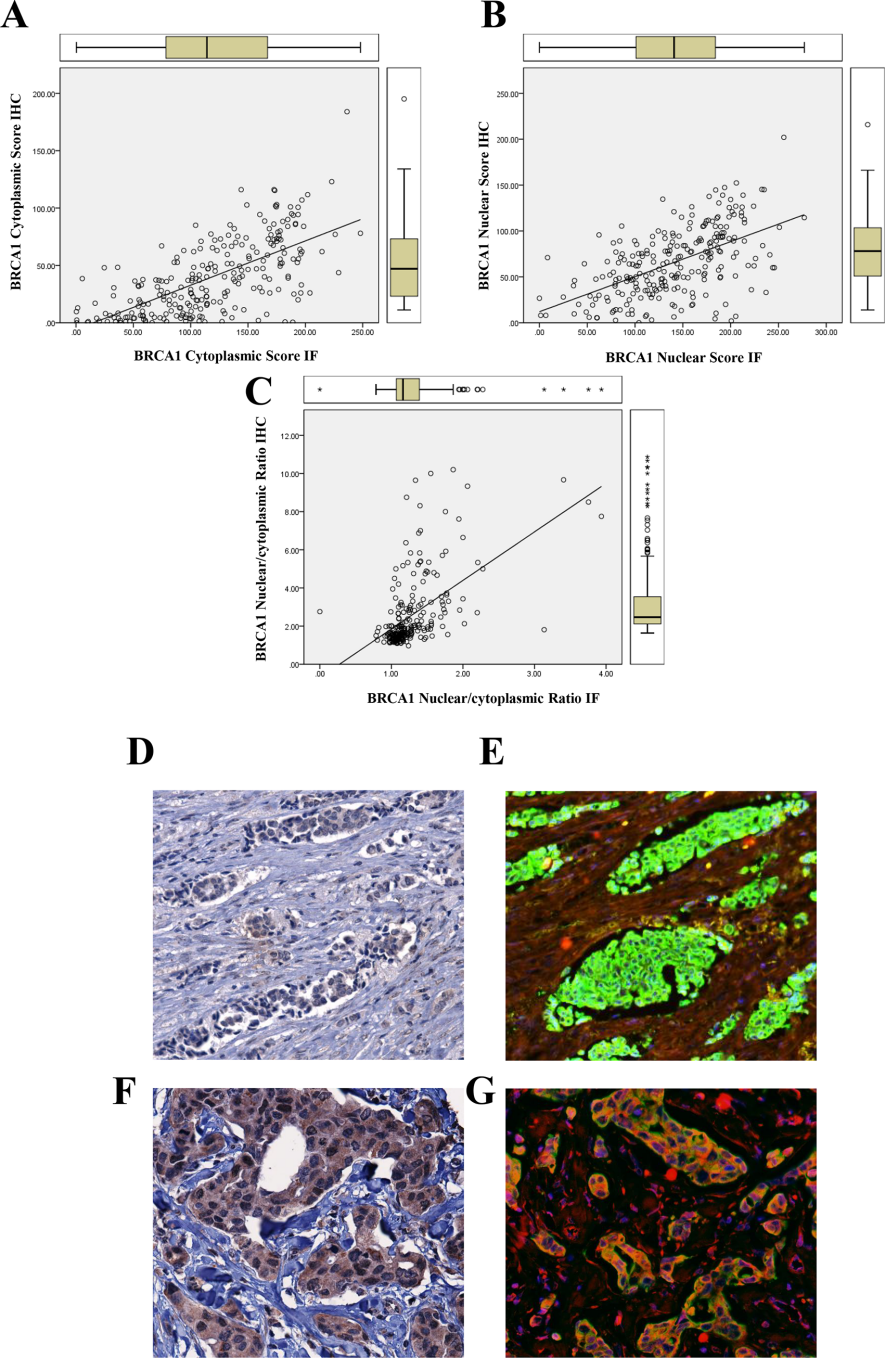
Dual IF vs IHC staining for BRCA1 analysis. Plot of pairwise dual IF versus IHC digital H-score for BRCA1 showing a good correlation between the two staining methods for cytoplasmic scores (A), nuclear scores (B), and nuclear/cytoplasmic ratios (C). Figures D to G demonstrate the correlation between the IHC staining (Figs. D and F) and the dual IF staining (Figs. E and G) in the corresponding cores.

IHC and IF staining scores were significantly correlated for cytoplasmic (r = 0.66, p = 0.000) (Fig 5A), and nuclear (r = 0.56, p = 0.000) (Fig 5B) BRCA1 expression as well as the N/C ratio (r = 0.55, p = 0.001) (Fig 5C), indicating a strong reliability between the two sets of measurements. Similar to the IF, IHC staining was distributed between the cytoplasm and the nuclear with a higher N/C ratio in breast cancer cases (2.9±0.2) than normal breast tissues (1.5±0.07, p = 0.001). BRCA1 IHC staining was lower in breast cancer cases than normal breast tissue in both the cytoplasmic (cancer: 39.2±1.9; Normal: 58.5±3.9, p = 0.037) and nuclear fractions (cancer: 65.4±2.3; normal: 81.8±4.4, p = 0.007) (Table 4). BRCA1 IHC staining was very weak in the stromal compartment in both normal breast and breast cancer tissues. Despite the weak IHC positivity and lower IHC H-scores compared with the IF staining, similar patterns of distribution and levels of expression were observed using the two methods (Fig 5D–5G).

**Table 4:** Summary of nuclear, cytoplasmic and N/C ratio of BRCA1 expression among invasive breast cancer samples (IF staining).

		Nuclear Score		Cytoplasmic score		Nuclear/Cytoplasmic	
Variable	N	Mean	P-value	Mean	P-value	Mean	P-value
Age							
<50 years	72	142		121		1.4	
50+ years	152	137		114		1.6	
Race							
nH White	80	143		118		1.5	
nH Black	106	144		122		1.6	
Hispanic	81	133		113		1.4	
Stage at diagnosis							
0	1	77		56		1.4	
1	103	143		119		1.3	
2	106	135		113		1.9	
3	35	150		126		1.3	
4	10	141		139		1.2	
Tumor grade			0.009		0.006		
Low	49	161		140		1.2	
Moderate	104	138		112		1.8	
High	97	132		112		1.4	
ER (H-Score)			0.046		0.14		
<10	78	123		101		1.2	
11–100	15	149		126		1.1	
101–200	43	138		120		1.5	
201–300	120	149		123		1.7	
PR (H-Score)							
<10	113	131		110		1.7	
11–100	27	140		116		1.3	
101–200	37	138		118		1.6	
201–300	85	145		119		1.4	
Her2			0.036		0.004		
Negative (ASCO/CAP score<3)	226	138		114		1.6	
Positive (ASCO/CAP score = 3)	37	158		142		1.1	
CK5/6			<0.0001		<0.0001		0.08
Negative (< 5% cells stain)	160	129		107		1.5	
Positive (≥5% cells stain)	97	162		138		1.2	
EGFR			0.04		0.04		
Negative (ASCO/CAP score<3)	237	139		116		1.6	
Positive (ASCO/CAP score = 3)	29	161		138		1.2	
Ki67					0.16		
Low (<14% cells stain)	147	139		113		1.7	
High (≥14% cells stain)	113	141		123		1.4	
P53							
Low (<20% cells stain)	196	139		116		1.6	
High (≥20% cells stain)	60	142		124		1.2	
BCL2							
Negative (<20% cells stain)	117	135		114		1.7	
Positive (≥20% cells stain)	142	143		119		1.4	
Nuclear AR			<0.0001		0.003		
Low (H-score<mean)	147	125		107		1.5	
High (H-score≥mean)	105	154		127		1.7	
Histological subtypes			0.16		0.006		<0.0001
Ductal	191	143		123		1.3	
Lobular	28	136		100		2.0	
Mixed ductal and lobular	19	115		84		3.9	
Others	15	145		132		1.1	
Molecular subtypes			0.08		0.019		
LuminalA	175	138		113		1.6	
LuminalB	14	174		156		1.1	
Her2	22	135		115		1.2	
Triple Negative	47	147		132		1.1	
Basal-like			<0.0001		<0.0001		0.08
No	155	128		106		1.7	
Yes	111	160		136		1.2	
Early family history			0.014		0.02		0.001
No	208	141		118		1.4	
Yes	14	104		83		3.8	

### Associations with patient and tumor characteristics

#### IF staining

**Fig 6:**
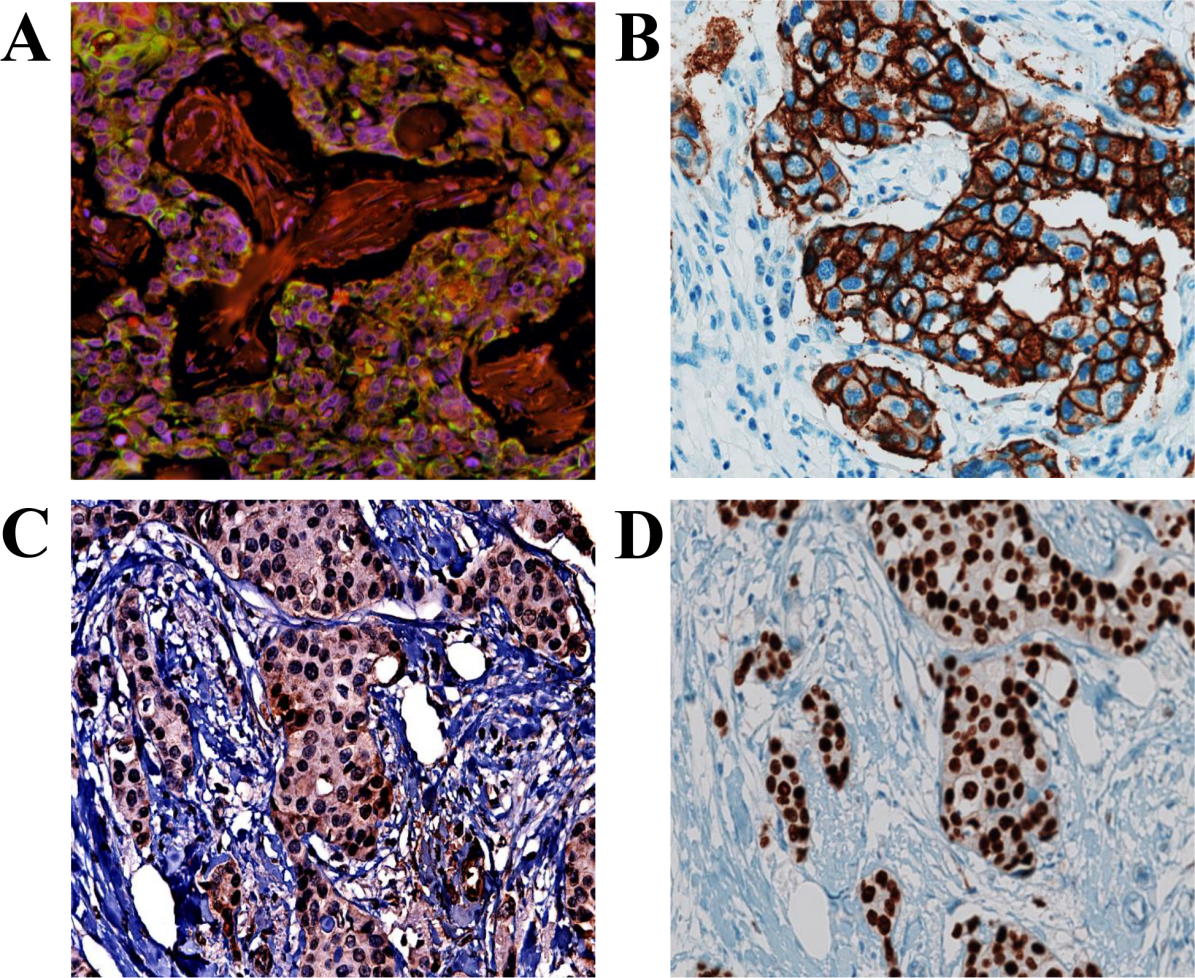
Association between BRCA1 and other prognostic markers. A: Positive BRCA1 staining in an invasive ductal carcinoma core. B, C, and D: Positive Her2, CK5/6 and AR staining in corresponding sections of the same core.

Associations of BRCA1 expression with patient, clinical and tumor characteristics were broadly similar between compartments (nuclear or cytoplasmic) (Table 4). Family history of breast cancer was associated with lower BRCA1 H-score with a higher fraction of BRCA1 protein is located in the nucleus (N/C ratio: positive family history = 3.8; no family history = 1.4; p = 0.001). BRCA1 H-score decreased as tumor grade increased (15% and 20% less in grade 2 and 3 than grade 1, respectively, p = 0.009) and was 25% to 30% higher for luminal B than other molecular subtypes (Table 4). When tumors were classified according to their basal nature using basal cell markers (CK5/6 and EGFR), basal-like breast cancer had significantly higher nuclear and cytoplasmic BRCA1 H-score (p<0.0001) and lowered N/C ratio than the non-basal tumors. Also, BRCA1 expression was higher among Her2 and nuclear AR-positive tumors (Fig 6).

Nuclear and cytoplasmic BRCA1 expression was then classified as low or high using the mean of the H-score as a cutoff (141 ± 3 and 118 ± 3, respectively). High BRCA1 expression was more frequent in luminal B subtype (86%; nuclear and cytoplasmic), followed by Her2 subtype (55% nuclear; 60% cytoplasmic) and was less frequent in luminal A and triple negative subtypes (Table 5). In basal-like breast cancer cases, 63% had high BRCA1 expression while only 37% had a low BRCA1 expression (χ2 = 11.6, p b = 0.001). In support of this finding, high nuclear BRCA1 expression was also associated with greater expression of the basal-like markers EGFR (χ2 = 4.3, p b = 0.04) and CK5/6 (χ2 = 10.4, p b = 0.001). High expression of BRCA1 (nuclear or cytoplasmic) was positively associated with high AR nuclear expression and low-grade tumors. Interestingly, the majority of cases with low BRCA1 N/C ratio (~60%) was associated with high ki67 LI (p b = 0.007) and basal nature of the tumor (p b = 0.02) (not tabulated).

**Table 5:** Associations between nuclear, cytoplasmic, and nuclear/cytoplasmic ratio of BRCA1 expression (IF staining) and clinicopathologic features in breast cancer.

Variable	Nuclear			Cytoplasmic		
	Low (%)	High (%)	P-value	Low (%)	High (%)	P-value
**Age**						
Young <50y	44.4	55.6		45.8	54.2	
Old > = 50y	53.3	46.7		53.3	46.7	
**Race**						
White non-Hispanic	46.3	53.8		48.8	51.3	
Black	49.1	50.9		49.1	50.9	
Hispanic	54.3	45.7		51.9	48.1	
**Tumor stage**			0.14			
Stage 0	100	0		100	0	
Stage 1	48.5	51.5		51.5	48.5	
Stage 2	56.6	43.4		51.9	48.1	
Stage 3	37.1	62.9		42.9	57.1	
Stage 4	30	70		30	70	
**Tumor grade**			0.05			0.025
Grade 1	38.8	61.2		32.7	67.3	
Grade 2	49	51		55.8	44.2	
Grade 3	57.7	42.3		51.5	48.5	
**ER**			0.004			
0 (H-score<10)	72.1	27.9		62.8	37.2	
1 (H-score = 11–100)	40	60		40	60	
2 (H-score = 101–200)	53.8	46.2		51.3	48.7	
3 (H-score = 201–300)	40.8	59.2		45.8	54.2	
**PR**						
0 (H-score<10)	59.5	40.5		54.1	45.9	
1 (H-score = 11–100)	51.9	48.1		48.1	51.9	
2 (H-score = 101–200)	52.2	47.8		51.3	48.7	
3 (H-score = 201–300)	44.7	55.3		48.2	51.8	
**Her2**			0.02			0.007
Negative (ASCO/CAP score<3)	53.1	46.9		53.5	46.5	
Positive (ASCO/CAP score = 3)	32.4	67.6		29.7	70.3	
**CK5/6**			0.001			0.002
Negative (< 5% cells stain)	56.9	43.1		57.5	42.5	
Positive (≥5% cells stain)	36.1	63.9		37.1	62.9	
**EGFR**			0.038		0.038
Negative (ASCO/CAP score<3)	51.5	48.5		51.5	48.5	
Positive (ASCO/CAP score = 3)	31	69		31	69	
**Ki67**						
Low (<14% cells stain)	50.3	49.7		53.7	46.3	
High (≥14% cells stain)	50.4	49.6		46	54	
**P53**						
Low (<20% cells stain)	50	50		51	49	
High (≥20% cells stain)	53.3	46.7		48.3	51.7	
**bcl2**						
Negative (<20% cells stain)	50.4	49.6		52.1	47.9	
Positive (≥20% cells stain)	51.4	48.6		49.3	50.7	
**Nuclear AR**			0.004			0.025
Low (H-score<mean)	59.2	40.8		57.1	42.9	
High (H-score≥mean)	41	59		42.9	57.1	
**Histological subtypes**						0.046
Ductal	49.2	50.8		46.1	53.9	
Lobular	46.4	53.6		64.3	35.7	
Mixed ductal and lobular	63.2	36.8		73.7	26.3	
Others	53.3	46.7		46.7	53.3	
**Molecular subtypes**			0.033			0.023
LuminalA	52.6	47.4		53.1	46.9	
LuminalB	14.3	85.7		14.3	85.7	
Triple Negative	57.4	42.6		57.4	42.6	
Her2	45.5	54.5		40.9	59.1	
**Basal-like**			0.001			0.002
No	58.1	41.9		57.4	42.6	
Yes	36.9	63.1		37.8	62.2	
**Early family history**			0.11			0.032
No	49	51		49	51	
Yes	71.4	28.6		78.6	21.4	

#### IHC staining

Continuous IHC nuclear score for BRCA1 correlated positively with CK5/6 (r = 0.24, p<0.0001), basal nature of the tumor (r = 0.52, p<0.0001), bcl2 (r = 0.13, p = 0.04), and AR nuclear (r = 0.33, p<0.0001) scores and negatively with age (r = -0.14, p = 0.048) and tumor grade (r = -0.25, p = 0.01) (Table 6). BRCA1 expression was then categorized into low and high using the mean value as a cutoff (Table 7). Some of the associations we reported above for the BRCA1 nuclear IF score were not evident for BRCA1 nuclear IHC score such as Her2 and molecular subtype. However, the association between high BRCA1expression and basal-like cancer (χ2 = 5.2, p b = 0.015) persisted. Reduced nuclear expression of BRCA1 was associated with higher tumor grades; grade 2 and 3 (60%) versus 42% in grade 1 (χ2 = 4.6, p b = 0.035). Low nuclear BRCA1 expression was also associated with negative or low bcl2 expression (χ2 = 5.4, p b = 0.014) and low nuclear AR expression (χ2 = 6.5, p b = 0.008)

**Table 6:** Summary of nuclear, cytoplasmic and N/C ratio of BRCA1 expression among invasive breast cancer samples (IHC staining).

		Nuclear Score		Cytoplasmic score		Nuclear/Cytoplasmic	
Variable	N	Mean	P-value	Mean	P-value	Mean	P-value
Age			0.048		0.08		0.09
<50 years	72	71		44		2.5	
50+ years	152	61		36		3.4	
Race							
nH White	80	68		40		3.4	
nH Black	106	65		40		2.8	
Hispanic	81	63		38		2.8	
Stage at diagnosis							
0	1	55		18		3.1	
1	103	66		40		2.8	
2	106	64		38		3.0	
3	35	64		38		3.3	
4	10	69		49		2.1	
Tumor grade			0.01		0.014		
Low	49	79		50		2.3	
Moderate	104	60		34		3.3	
High	97	63		39		2.9	
ER (H-Score)							
<10	78	59		35		2.8	
11–100	15	57		34		2.8	
101–200	43	66		39		3.9	
201–300	120	70		43		2.8	
PR (H-Score)			0.08		0.15		0.1
<10	113	62		37		2.9	
11–100	27	59		34		4.4	
101–200	37	62		35		3.2	
201–300	85	64		46		2.5	
Her2					0.17		
Negative (ASCO/CAP score<3)	226	64		38		3.1	
Positive (ASCO/CAP score = 3)	37	73		46		2.7	
CK5/6			<0.0001		0.001		0.014
Negative (< 5% cells stain)	160	59		34		3.4	
Positive (≥5% cells stain)	97	77		48		2.3	
EGFR			0.07		0.06		
Negative (ASCO/CAP score<3)	237	64		38		3.1	
Positive (ASCO/CAP score = 3)	29	77		50		2.5	
Ki67			0.09		0.03		0.03
Low (<14% cells stain)	147	63		37		3.2	
High (≥14% cells stain)	113	70		44		2.4	
P53					0.12		0.09
Low (<20% cells stain)	196	65		38		3.1	
High (≥20% cells stain)	60	69		44		2.4	
BCL2			0.04		0.08		
Negative (<20% cells stain)	117	61		35		3.3	
Positive (≥20% cells stain)	142	69		44		2.7	
Nuclear AR			<0.0001		0.001		0.06
Low (H-score<mean)	147	58		34		3.2	
High (H-score≥mean)	105	76		47		2.5	
Histological subtypes					0.12		
Ductal	191	67		41		3.0	
Lobular	28	64		31		3.4	
Mixed ductal and lobular	19	50		26		3.2	
Others	15	68		46		2.0	
Molecular subtypes			0.15		0.046		
LuminalA	175	65		38		3.1	
LuminalB	14	88		63		2.4	
Her2	22	63		35		2.8	
Triple Negative	47	65		39		2.8	
Basal-like			<0.0001		<0.0001		0.01
No	155	58		33		3.4	
Yes	111	76		48		2.4	
Early family history							
No	208	65		39		3.1	
Yes	14	58		34		2.7	

**Table 7:** Associations between nuclear, cytoplasmic, and nuclear/cytoplasmic ratio of BRCA1 expression (IHC staining) and clinicopathologic features in breast cancer.

Variable	Nuclear			Cytoplasmic		
	Low (%)	High (%)	P-value	Low (%)	High (%)	P-value
**Age**			0.09			0.048
Young <50y	44.1	55.9		47.1	52.9	
Old > = 50y	54.8	45.2		60.3	39.7	
**Race**						
White non-Hispanic	50	50		56.4	43.6	
Black	50	50		57.5	42.5	
Hispanic	53.8	46.2		54.5	45.5	
**Tumor stage**						
Stage 0	100	0		100	0	
Stage 1	52.6	47.4		56.7	43.3	
Stage 2	49.5	50.5		57.3	42.7	
Stage 3	55.6	44.4		54.3	45.7	
Stage 4	50	50		50	50	
**Tumor grade**			0.035			0.09
Grade 1	35.6	64.4		42.2	57.8	
Grade 2	58.4	41.6		60.4	39.6	
Grade 3	53.6	46.4		59.4	40.6	
**ER**			0.11			
0 (H-score<10)	73.3	26.7		73.3	26.7	
1 (H-score = 11–100)	52	48		54.7	45.3	
2 (H-score = 101–200)	56.8	43.2		61.4	38.6	
3 (H-score = 201–300)	44.1	55.9		51.7	48.3	
**PR**			0.19			
0 (H-score<10)	57.5	42.5		62.2	37.8	
1 (H-score = 11–100)	51.9	48.1		55.6	44.6	
2 (H-score = 101–200)	51.4	48.6		59.4	40.6	
3 (H-score = 201–300)	41.7	58.3		48.8	51.2	
**Her2**						
Negative (ASCO/CAP score<3)	52.1	47.9		57.1	42.9	
Positive (ASCO/CAP score = 3)	45.7	54.3		51.4	48.6	
**CK5/6**						0.013
Negative (< 5% cells stain)	55.8	44.2		61.5	38.5	
Positive (≥5% cells stain)	43.2	56.8		46.3	53.7	
**EGFR**			0.12			0.044
Negative (ASCO/CAP score<3)	52.6	47.4		58.3	41.7	
Positive (ASCO/CAP score = 3)	39.3	60.7		39.3	60.7	
**Ki67**			0.10			0.017
Low (<14% cells stain)	57.3	42.7		61.5	38.5	
High (≥14% cells stain)	41.7	58.3		47.2	52.8	
**P53**						
Low (<20% cells stain)	52.6	47.4		57.3	42.7	
High (≥20% cells stain)	47.5	52.5		52.5	47.5	
**bcl2**			0.014			0.017
Negative (<20% cells stain)	59.3	40.7		63.7	36.3	
Positive (≥20% cells stain)	44.7	55.3		49.6	50.4	
**Nuclear AR**			0.008			0.03
Low (H-score<mean)	58	42		61.5	38.5	
High (H-score≥mean)	41.4	58.6		48.5	51.5	
**Histological subtypes**						
Ductal	49.7	50.3		53.8	46.2	
Lobular	58.6	41.4		69	31	
Mixed ductal and lobular	64.7	35.3		64.7	35.3	
Others	38.5	61.5		46.2	53.8	
**Molecular subtypes**						
LuminalA	50.6	49.4		57	43	
LuminalB	28.6	71.4		35.7	64.3	
Triple Negative	57.1	42.9		55.6	44.4	
Her2	55.6	44.4		61.9	38.1	
**Basal-like**			0.015			0.003
No	57	43		63.8	36.2	
Yes	42.6	57.4		45.4	54.6	
**Early family history**						
No	51.7	48.3		56.2	43.8	
Yes	50	50		58.3	41.7	

IHC cytoplasmic scores for BRCA1 were positively correlated with CK5/6 (r = 0.25, p = 0.000), Ki67 (r = 0.16, p = 0.01), and nuclear AR (r = 0.32, p = 0.000). BRCA1 cytoplasmic expression was then classified as high or low using the mean of H-score as a cutoff. High cytoplasmic BRCA1 expression was positively associated with basal like cancer (χ2 = 8.6, P b = 0.003) and basal cell markers CK5/6 (χ2 = 5.5, P b = 0.013) and EGFR (χ2 = 4.5, P b = 0.04) as well as Ki67 index (χ2 = 6.0, P b = 0.017), bcl2 (χ2 = 5.0, P b = 0.017), and nuclear AR expression (χ2 = 4.1, P b = 0.03).

In summary, our data demonstrated a significant positive association between high nuclear BRCA1 expression and basal cell nature of breast cancer tissues. A positive association was also found between nuclear BRCA1 and nuclear AR expression. There was a trend towards having high grade tumor, negative hormone receptors, low bcl2 expression and high Ki67 index in cases with low nuclear BRCA1 expression. BRCA1 expression tended to be lower in older age and in patient with positive family history however, the above-mentioned associations (with basal markers, tumor grade, AR, hormone receptors, bcl2 and Ki67 index) persisted after adjusting for age at diagnosis and family history. BRCA1 N/C ratio was significantly higher in mixed ductal and lobular carcinoma than other histological subtypes (p<0.0001) when BRCA1 was quantified using dual IF method. The latter association persisted after adjusting for age, family history, tumor grade, and ER status yet, such association was not detected using the IHC method. Finally, there was no significant association between BRCA1 expression and race or tumor stage at time of diagnosis using either the dual IF or the IHC methods.

## Discussion

The identification of BRCA1 mutations and their involvement in breast cancer have gained a lot of attention in the past decade. Far less understood, however, is BRCA1 expression and subcellular distribution in breast cancer tissues. Previous experimental studies have suggested that mechanisms other than mutations (e.g. epigenetic modifications) may alter BRCA1 expression and/or subcellular distribution in breast cancer [16]. This study has investigated BRCA1 protein expression and subcellular localization, and their relation to clinicopathological characteristics in a population-based study of an ethnically diverse sample of breast cancer patients using two different staining methods, IHC versus dual IF, and automated digital microscopy analysis.

Per our data, a high uniform expression of BRCA1 was observed in normal breast tissue while absent or reduced expression was found only in malignant tissues which is consistent with a previous study by Rakha *et al* [17] who reported either complete loss or reduced nuclear expression of BRCA1 in 54% of breast cancer cases. Our results also demonstrated that reduced BRCA1 expression is associated with more aggressive characteristics such as high grade and hormone receptor negative tumors and high proliferation index (Ki67). These findings are supported by previous studies that reported an association between altered BRCA1 expression and poor prognostic parameters [18, 19]. However, it is worth mentioning that some previous studies failed to demonstrate such association between BRCA1 protein expression and other prognostic features [20, 21], which could be the result of population heterogeneity or technical issues in achieving accurate measurements of BRCA1 [6].

BRCA1 protein is classified as a tumor suppressor gene that maintains genomic integrity via regulating DNA replication, repair, and transcription as well as various cell cycle checkpoints. To perform these functions, BRCA1 protein accumulates inside the nucleus. Previous studies have detected a shift in BRCA1 from nucleus to cytoplasm in breast cancer. Accordingly, we sought to examine whether reduced nuclear localization (measured as the ratio of nuclear to cytoplasmic expression) was associated with more aggressive tumor features. Our data demonstrated that BRCA1 nuclear to cytoplasmic (N/C) ratios were significantly lower in breast cancer than normal breast tissues. Lower BRCA1 N/C ratio was associated with early family history, basal-like nature of the tumor, and a high proliferation index (Ki67). There was a heterogeneity among previous studies with respect to BRCA1 subcellular distribution. For example, Taylor *et al*. [22] reported cytoplasmic localization of BRCA1 in breast cancer tissues while Wilson *et al*. [10] detected BRCA1 staining mainly in the nucleus. Yet, an association between low BRCA1 N/C ratio and high grade tumors and poor prognosis has been a consistent finding in many studies [17, 23, 24].

Although this association might indicate an influence of BRCA1 cytoplasmic retention on tumor progression, the mechanism and direction of causality in this association are uncertain. Different findings with respect to BRCA1 subcellular distribution and its prognostic value across studies could be the result of detecting different variants of BRCA1 protein among studies that use various antibodies and labeling methods. Although it has been thought that commercially available BRCA1 antibodies lack the specificity needed to identify BRCA1 protein [25], many studies concluded that the specificity of some of the BRCA1 antibodies is adequate to consider IHC as a valuable screening method [26]. Herein, we used two distinct methods of BRCA1 labeling (IHC versus dual IF) and two different antibodies one of which is MS110 (AB-1) that has been shown to be the most reliable, accurate, and reproducible antibody for immunolocalization of BRCA1 on breast cancer paraffin-embedded tissues [25]. The results from both settings support the association between low nuclear BRCA1 detection and poor prognostic indicators such as older age, higher tumor grades, weak ER and Her2 expression, and high proliferation index as well as positive family history of breast cancer.

In the current study, we also observed a strong correlation between high BRCA1 expression and the basal cell nature of the tumor (positive for EGFR and/or CK5/6). This observed link between BRCA1 expression and basal-like breast cancer in our study is supported by similar findings from previous studies showing that the majority of BRCA1-associated tumors express basal cytokeratins. However, unlike these studies, we could not find a significant association between BRCA1 staining and P53, a marker that is commonly seen in basal-like tumors [27–29]. Thus, in combination with evidence from previous studies, our data highlight basal cell markers as a potential predictor of altered BRCA1 expression that may be useful in selecting patients for BRCA1 mutation testing in routine practice. However, this needs to be confirmed by further studies where BRCA1 mutation status is available and directly correlated with basal cell markers.

Our data showed a trend towards having lower ER and PR immunoreactive scores in tumors that have low BRCA1 expression which is in agreement with previous studies showing BRCA1-related breast cancers are more frequently ER negative than nonhereditary breast cancers [30]. These findings can be explained by the fact that ER promoter activity and transcription are regulated by the nuclear fraction of BRCA1 protein [31] and that several BRCA1 mutations interfere with BRCA1 nuclear import resulting in disturbed BRCA1 nuclear function [32]. Yet, impaired BRCA1 nuclear import has been also detected in sporadic non-familial cases [22]. A growing body of experimental evidence suggests a role of BRCA1 chaperones such as BARD1 and BRAP2 which regulate BRCA1 nuclear localization and cytoplasmic retention, respectively [8]. It is conceivable that any disturbances in the expression or function of these chaperones will influence BRCA1 nuclear localization and subsequently its transcriptional activity even in the absence of BRCA1 mutations. However, further verification of this mechanism and its implications in familial and sporadic breast cancer remain to be explored.

We also found that Her2 positive tumors are significantly associated with high BRCA1 expression. Accordingly, Luminal B and Her2-enriched molecular subtypes had higher BRCA1 scores than Luminal A and triple negative tumors. Previous studies have reported low HER2 amplification amongst BRCA1 mutation carriers who conceivably have impaired BRCA1 expression and/or function [33]. These findings might indicate a connection between BRCA1 and Her2 pathways in breast cancer that is similar to what have been shown for BRCA1 and ER except that the mechanistic link between BRCA1 and Her2 is less understood.

One of the significant and novel findings in this study is the strong positive correlation between BRCA1 and AR expression. AR has been reported to be expressed in 60–90% of sporadic breast cancer and is considered an independent prognostic factor of better outcome in ER-positive patients [34]. AR has been shown to be expressed in 80% of BRCA2 mutated breast cancer as opposed to only 30% of BRCA1 mutated breast cancer [35]. To our knowledge, this study is one of only two studies (Rakha *et al* [17]) that measured the association between AR and BRCA1 protein expression in a population-based study of breast cancer patients regardless of BRCA1 mutation status (i.e. both hereditary and sporadic cases). Park *et al*. [36] demonstrated that BRCA1 is a coactivator of AR and might directly modify AR signaling and subsequently AR autoregulated expression *in vitro*. Furthermore, other studies have demonstrated that AR signaling stimulates the expression of tumor suppressor genes such as PTEN, resulting in cell growth inhibition and activation of p53-mediated apoptosis in breast cancer [37]. Whether BRCA1 is upstream or downstream to AR signaling is not completely understood. Yet, the strong positive correlation between AR and BRCA1 should inspire future studies to explore AR signaling as a therapeutic target in familial breast cancers that have altered BRCA1 expression.

An important role for BRCA1 expression and subcellular localization in breast cancer progression is indicated by its loss and altered distribution in a large proportion of non-familial breast cancers. Yet, the speed and low price of current BRCA1 mutation screening make it a more popular method to assess BRCA1 status in breast cancer than measuring the actual expression and subcellular distribution of BRCA1 in histological specimens. However, BRCA1 mutation screening is helpful only for individuals who receive a clear-cut result (pathogenic mutation with a known function or negative for any mutation) and not for others who have BRCA1 mutations with uncertain significance. Our data indicated that BRCA1 expression and subcellular localization might stand as an independent prognostic factor in breast cancer which is supported by similar findings from previous studies [17, 23, 38]. However, this research would benefit greatly from larger population-based studies that might encourage health care workers to incorporate BRCA1 staining in breast cancer prognosis algorithm.

Major strengths of this study are that it is a population-based study of an ethnically diverse sample of breast cancer patients and therefore may be generalizable to an urban population. Another strength was the availability of detailed demographic and clinical data. Also, using dual IF technique and automated digital image analysis allowed to segment tissue compartments (epithelium vs. stromal) and subcellular compartments (nucleus versus cytoplasm) and thus enabled accurate per cell quantification of BRCA1 defined within multiple tissue types including epithelium and stroma. It also allowed the quantitation of weakly expressing and overlapping biomarkers within cytoplasm and nuclei which cannot be identified by the naked eye, ensuring consistency of scoring and minimizing manual error. The strong correlation between the dual IF staining and the IHC staining using the widely used MS110 antibody validate the former method as an alternative approach that exhibits a stronger signal in the epithelial compartment and avoids contamination by stromal non-specific positivity. Yet, the cross-sectional nature of this study hinders the ability to assess temporal aspects of the observed associations. There are also limitations in the tissue microarray technique used in this and other studies. Tissue stained might not be representative of the tumor due to tumor heterogeneity. However, to avoid this, TMA blocks were constructed in triplicates, each containing one sample from a different region of the tumor. Also, many groups have shown excellent concordance between tissue microarray spots and whole sections in IHC studies of multiple tumor types [39, 40]. Finally, information about BRCA1 mutation status in the study subjects is not available which would have strengthened the validity of our current findings. Nevertheless, the main purpose of the current study was to examine the expression and localization of BRCA1 protein and to assess its prognostic value, in an ethnically diverse sample of breast cancer patients that represents the major racial/ethnic groups in Chicago, independent of BRCA1 gene mutations.

In conclusion, an important role for the BRCA1 protein in breast cancer progression is indicated by its reduction or altered subcellular distribution in a large proportion of breast cancer patients. Correlations between reduced BRCA1 expression and some aggressive tumor features in our study were, to some extent, similar to those described in previous studies of familial cancers with BRCA1 germline mutation [5]. Research on a more basic level is required to understand the underlying genetic variants that different BRCA1 assays are identifying, and whether commercially available antibodies may select preferentially for proteins derived from cytoplasmic variants or vice versa. Only then might we consider adding BRCA1 as a clinical tool in assessing prognosis.
